# Sexual dimorphism of stress response and immune/ inflammatory reaction: the corticotropin releasing hormone perspective

**DOI:** 10.1155/S0962935195000275

**Published:** 1995-05

**Authors:** Nicholas V. Vamvakopoulos

**Affiliations:** 1 Department of Biology-Genetics University of Thessaly Medical School Larisa 412 22 Greece

## Abstract

This review higlghts key aspects of corticotropin releasing hormone (CRH) biology of potential relevance to the sexual dimorphism of the stress response and immune/inflammatory reaction, and introduces two important new concepts based on the regulatory potential of the human (h) CRH gene: (1) a proposed mechanism to account for the tissue-specific antithetical responses of hCRH gene expression to glucocorticolds, that may also explain the frequently observed antithetical effects of chronic glucocorticoid administration in clinical practice and (2) a heuristic diagram to illustrate the proposed modulation of the stress response and immune/ inflammatory reaction by steroid hormones, from the perspective of the CRH system.


Mediators of Inflammation 4, 163–174 (1995)

### Sexual dimorphism of stress response and immune/ inflammatory reaction: the corticotropin releasing hormone perspective

Nicholas V. Vamvakopoulos

Department of Biology-Genetics, University of Thessaly Medical School, 412 22 Larisa, Greece

**THIS review highlights key aspects of corticotropin releasing hormone (CRH) biology of potential relevance to the sexual dimorphism of the stress response and immune/inflammatory reaction, and introduces two important new concepts based on the regulatory potential of the human (h) CRH gene: (1) a proposed mechanism to account for the tissue-specific antithetical responses of hCRH gene expression to glucocorticoids, that may also explain the frequently observed antithetical effects of chronic glucocorticoid administration in clinical practice and (2) a heuristic diagram to illustrate the proposed modulation of the stress response and immune/inflammatory reaction by steroid hormones, from the perspective of the CRH system.**

**Key words:** Autoimmunity, Corticotropin releasing hormone, Estrogens, Glucocorticoids, Glucocorticoid receptor, Gonadal steroids, Heat shock proteins, Hormonal regulation of gene expression, Hypothalamic–pituitary–adrenal axis, Immune/inflammatory reaction, Sexual dimorphism, Stress response

### Introduction

Experimental support for the hypothesis that adrenocorticotropin (ACTH) secretion was controlled by hypothalamic factors,[1] was obtained in 1955.[2,3] In 1981, a 41 amino acid C terminal amidated peptide from ovine hypothalami stimulating pituitary ACTH release *in vitro* was identified and characterized.[4] The biologically active form of this peptide, designated corticotropin releasing hormone (CRH), and also frequently referred to as corticotropin releasing factor (CRF), was synthesized and found to have potent ACTH-releasing actions *in vivo*.[5] CRH is the only permissive factor for the anterior pituitary release of ACTH known in man[5,6] and acts in synergy with arginine vasopressin (AVP) and, perhaps, other factors, to regulate pituitary ACTH secretion, and, therefore ultimately the activity of the pituitary–adrenal axis.[7–9]

Since its discovery, it has become evident that CRH has roles which are much wider than initially thought. Thus, coordination of the behavioral and physical components of the stress response and regulation of the immune/inflammatory reaction were unravelled as major overall roles of this neuropeptide.[10,11] In addition, this peptide was implicated in the pathophysiology of a large range of diseases associated with dysregulation of the stress system and autoimmunity.[12] Because of the central roles of CRH in homeostasis and pathogenesis of disease, knowledge of its gene and its regulation would be essential for further progress. This brief review will outline the most critical aspects of CRH biology, will summarize the structure, function and regulation of the human (h) CRH gene by steroid hormones, and will introduce a tentative model to account for its tissue-specific antithetical hormonal responses. The implications of the regulatory potential of the hCRH gene for the sexual dimorphism of the stress response and immune/inflammatory reaction will also be discussed.

### Overview of CRH biology

CRH is synthesized as part of a prohormone, it is processed enzymatically, and in addition undergoes enzymatic modification to the amidated form. Mammalian CRH has homologies with non-mammalian vertebrate peptides x CRH[13] and sauvagine[14] in amphibia (from frog brain/spleen and skin, respectively), urotensin-I in teleost fish[15] and the two diuretic peptides Mas-DPI and Mas-DPII from the tobacco hornworm *Manduca sexta*.[16,17] The vertebrate homologues have been tested and found to possess potent mammalian and fish pituitary ACTH-releasing activity. In addition, they decrease peripheral vascular resistance and cause hypotension when injected into mammals.[15,18,19]

The amino terminus of CRH is not essential for binding to the receptor, whereas absence of the carboxy terminal amide abolishes CRH binding to

© 1995 Rapid Communications of Oxford Ltd its receptor. Oxidation of a methionine residue abolishes the biological activity of CRH, and this may be a mechanism for neutralization of the peptide *in vivo*.[5] CRH bioavailability is also regulated by binding to corticotropin releasing hormone binding protein (CRHBP),[20] with which it partially co-localizes in the rat central nervous system (CNS) and other tissues.[21] The human CRHBP gene has been assigned to 5q11.2–q13.3.[22] In the pituitary, CRH acts by binding to membrane receptors (CRHR) on corticotrophs, that couple to guanine nucleotide-binding proteins and stimulate the release of ACTH in the presence of $Ca^{2+}$ by a cAMP-dependent mechanism.[5,23,24] CRH stimulation of cAMP production increases in parallel with the secretion of ACTH in rat pituitary corticotrophs[25] and human corticotroph cells.[26] In addition to enhancing the secretion of ACTH, CRH also stimulates the *de novo* biosynthesis of pro-opiomelanocortin (POMC).[25,27] CRH regulation of POMC gene expression in mouse tumorous corticotroph AtT 20 cells, involves the induction of c-fos expression by cAMP and $Ca^{2+}$-dependent mechanisms.[28,29]

Sequence analysis of hCRHR cDNAs isolated from cDNA libraries prepared from human corticotropinoma or total human brain mRNA, revealed homology to the G-protein coupled receptor superfamily.[30,31] The hCRHR cDNA sequences of the tumour and normal brain were aligned and found to be identical. The hCRHR gene has been assigned to 17q12–qter.[32] The sequences of mouse and rat CRHR cDNAs were also reported recently.[31,33] Human/rodent CRHR protein sequences differ primarily in their extracellular domains. In particular, positively charged arginine amino acid(s) are present in the third and fourth positions of the extracellular amino terminal domain of the rodent but not the human CRHR peptide. This might be responsible for the differential activity of the α-helical 9-41 CRH antagonist[34] between rodents and primates (C. Kalogeras, personal communication).

Central sites of CRHR expression include the hypothalamus, the cerebral cortex, the limbic system, the cerebellum and the spinal cord.[35] This may explain the broad range of neural effects of intracerebroventricularly (i.c.v.) administered CRH, including arousal, increase of sympathetic system activity, elevations in systemic blood pressure, tachycardia, suppression of the hypothalamic component of gonadotropin regulation (GnRH), suppression of growth and inhibition of feeding and sexual behaviours characteristic of emotional stress.[36–42] Central injection of CRH in rats and monkeys thus induces complex physiological and behavioral responses, suggesting that central CRH pathways coordinate the overall stress response.[40] High doses of CRH cause behaviors characteristic of anxiety, suggesting that the behavioral effects of CRH are dose-dependent, with low doses promoting adaptation and high doses being maladaptive.[12,42]

There is a broad peripheral expression of CRH and CRHR, including the peripheral nervous system, lung, liver, gastrointestinal tract, immune cells and organs, gonads and placenta.[10,11,43] The biological roles of extraneural CRH have not been fully elucidated as yet, although it is likely that it might participate in the auto/paracrine regulation of β-endorphin production and analgesia, and that it may modulate immune/inflammatory responses and gonadal function.[12,41,44–46] The current consensus is that CRH produced in high amounts in inflammatory sites of both animals and humans, designated immune CRH, is promoting inflammation by stimulating cytokine production by immune cells and/or by potentiating the proinflammatory activities of cytokines and other mediators of inflammation.[46,47–53]

Intravenous administration of CRH in humans causes a prompt increase in the release of ACTH into the blood, followed by the secretion of cortisol. The effect is specific for ACTH release and is inhibited by glucocorticoids. High cortisol levels reduce or abolish CRH action on the pituitary. CRH has been used as a diagnostic tool to differentiate causes of hypercortisolism and hypocortisolism, but does not have an established therapeutic role.[24,54–56] The clinical applications of CRH were recently reviewed extensively.[11]

The amidated active peptide form is stored within secretory granules. Stress stimulates a variety of endogenous substances, which excite the CRH neuron in the PVN[12] and cause the release of CRH into the portal system by the classical mechanism of membrane fusion.[57] Major intracellular signalling systems, such as the cyclic adenosine monophosphate (cAMP)-dependent protein kinase A (PKA) [cAMP/PKA] and the diacyglycerol (DAG)-dependent protein kinase-C (PKC) [DAG/PKC] pathways, appear to be involved in the regulation of CRH biosynthesis and release.[58] Theoretically, hormonal regulation of CRH biosynthesis and secretion, and CRHR-mediated signal transduction may occur in any of many steps.[10]

### hCRH gene structure and chromosomal localization

The hCRH gene consists of two exons separated by an intron in its 5′ untranslated region (Fig. 1).[59] The rat and ovine CRH genes have a similar organization.[60,61] The hCRH gene has

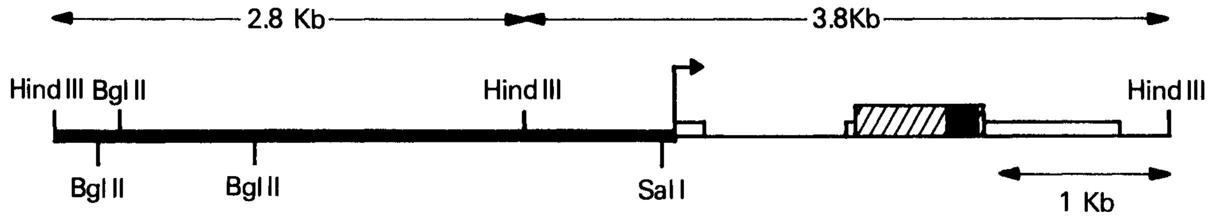

FIG. 1. Physical organization of a 6.6 kb DNA region containing the hCRH gene. The bent arrow marks the start and points towards the direction of transcription. The open boxes refer to the transcribed and untranslated mRNA sequences, the striped box to the translated prepro-hCRH mRNA and the small black box to the segment of the gene, coding the hCRH(1-41) peptide, which is derived from the precursor hCRH protein by post-translational proteolytic cleavage. The 5'-flanking region of the gene is indicated by a thick black line.

been mapped to the long arm of chromosome 8 (8q13).[62] The 3′ untranslated region of the hCRH gene contains several polyadenylation sites, which may be utilized differentially in a potentially tissue-specific manner. CRH mRNA polyA tail length is regulated by phorbol esters in the human hepatoma CRH-expressing cell line NPLC, and this may have potential relevance for differential stability of CRH mRNA in various tissues *in vivo*.[63] Alignment of the human, rat and ovine CRH gene sequences has allowed the comparison of the relative degree of evolutionary conservation of their various segments. These comparisons revealed that the 330 bp long proximal segment of the 5′ flanking region of the hCRH gene had the highest degree of homology (94%), suggesting that it may play a very important role in CRH gene regulation throughout phylogeny, which is crucial for survival. A conserved polypurine sequence feature of unknown biological significance is present at −829 of hCRH, −801 of the ovine CRH gene, as well as in the −400 bp 5′ flanking region of POMC, rat growth hormone and other hormone genes.[64] The sequence of a 3.7 kb stretch into the 5′ flanking region of the hCRH gene was also determined (Gene Bank accession no. x67661).[65] A gene bank search for homologous sequences identified a segment at position 2213–2580 with greater than 80% homology to members of the type O family of repetitive elements, and another at −2835 to −2972 with less homology to the 3′ terminal half of the human AluI family of repetitive elements. The rest of the sequence was found to be novel and specific to the CRH gene. A computer program was used to identify consensus recognition sites of DNA binding proteins involved in transcriptional regulation in this region of the hCRH gene. The organization and spatial distribution of several putative responsive elements of potential relevance to CRH transcriptional regulation is shown in Fig. 2.[65]

### Regulatory elements of the hCRH gene

*CRE (cAMP-responsive enhancer, CREB enhancer sequence/PKA activation)*: Activation of adenylate cyclase by various effector systems elevates intracellular cAMP and stimulates protein phosphorylation by cAMP-dependent protein kinases and transactivation of genes with the consensus palindromic sequence 5′-TGACGTCA-3′ in their 5′ flanking regions.[66] cAMP-response element-binding protein (CREB), is a member of the bZIP or leucine zipper family of transcription factors, that is phosphorylated by several protein kinases, including the catalytic subunit of cAMP/PKA. CREB homo- or heterodimerizes through its leucine zippers, binds to DNA as a dimer, and modulates transcription of genes in response to hormonal stimulation of the cAMP pathways.[67–70] The gene encoding CREB contains at least 11 exons spanning over 40 kb and produces multiple CREB isoforms by alternative splicing.[71,72] This is in contrast to other bZIP members (e.g., c-jun[72] and C/EBP[73]), which are encoded by intronless genes.[74] The latter proteins can heterodimerize with some members of the CREB/ATF family of proteins.[75,76]

CRH regulation by the PKA pathway is well documented. Administration of cAMP increases CRH secretion from perfused rat hypothalami, and forskolin, an activator of adenylate cyclase, increases CRH secretion and CRH mRNA levels in primary cultures of rat hypothalamic cells.[77] Regulation of the hCRH gene by cAMP has also been demonstrated in the mouse tumorous anterior pituitary cell line AtT-20, stably or transiently transfected with the hCRH gene.[78–80] The hCRH 5′ flanking sequence contains a perfect consensus CRE element that is conserved in the rat and sheep, and confers transcriptional activation to chloramphenicol acetyltransferase (CAT) reporters *in vivo*, as demonstrated with both human and rat CRH promoter-driven CAT constructs.[81,82]

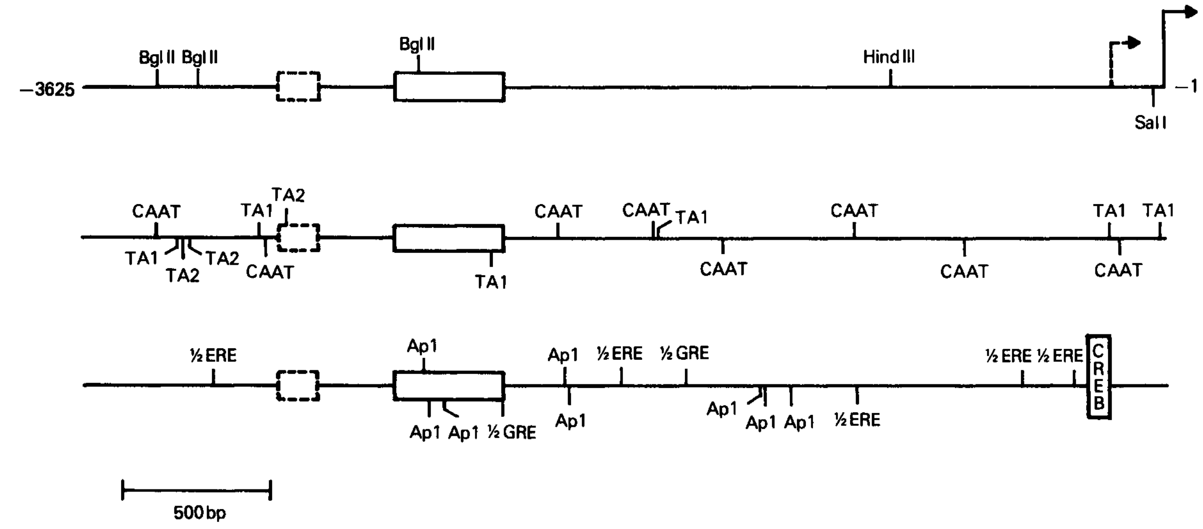

FIG. 2. Spatial distribution of putative regulatory features in the 3625 bp long hCRH 5′ flanking region. *Top*: landmark restriction sites, the two mRNA initiation sites (solid and broken bent arrows point to the direction of transcription), the type O member of repetitive elements (solid box), and the partial AluI member of repetitive elements (broken box). *Middle*: locations of TATA box 1 [TA1: 5′TATAAA(+), 5′TTTATA(–)]; TATA box 2 [TA2: 5′TATAAT(+), 5′ATTATA(–)]; and CAAT box [5′CCAAT(+), 5′ATTGG (–)] consensus elements. *Bottom*: locations of CREB [5′ACGTCA(+), 5′TGATTCA(–), 5′TGACGTCA(+), 5′AGACGTCA(–)]; AP-1 [5′TGAAATCA(+), 5′TGATTCA(–), 5′TGAGACTT(+), 5′AAGTCTCA(–), 5′TGACTAA(+), 5′TTAGTCA(–), 5′TTAGTCAG(+), 5′CTGACTAA(–), 5′CTGACTAA(+), 5′TTAGTCAG(–); half palindromic ER sites (1/2 ERE) [5′GGTCA(+), 5′TGACC(–)]; and second half GR site (1/2 GRE) [5′TGTTCT(+), 5′AGAACA(–)] consensus elements. The (+) and (–) orientations of the elements are indicated by the arrow pointing above or below the midline, respectively.

*TRE (phorbol ester, 12-O-tetradecanoyl phorbol-13-acetate (TPA)- response element or AP-1 site/ PKC activation)*: Epidermal growth factor (EGF) and TPA elevate intracellular DAG and activate PKC and the transactivation of genes containing TPA response elements in their 5′ flanking regions.[83] TRE or AP-1-binding proteins are the Jun and Fos families of the bZIP superfamily of transcription factors. These proteins interact through their carboxy-terminal leucine zippers and c-jun can form both homodimers with itself and heterodimers with c-fos, while c-fos can heterodimerize with c-jun but cannot form homodimers. TPA and the activation of PKC induce c-jun. This is due to positive autoregulation mediated by binding of c-jun homodimers or c-jun:c-fos heterodimers to an AP-1 site within the c-jun promoter.[84,85]

TPA, an activator of PKC, stimulates CRH mRNA levels and peptide secretion by 1.5- to 2-fold in primary cultures of rat hypothalamic cells.[77] TPA also increases CRH mRNA levels by almost 16-fold and CRH mRNA poly-A tail length by about 100 nucleotides in the human hepatoma cell line NPLC.[63] The proximal 0.9 kb 5′ flanking the hCRH gene confers TPA inducibility to a CAT reporter in transient expression assays.[86] In the absence of a clearly discernible perfect TRE in this region, it has been suggested that the CRE of the CRH promoter may, under certain conditions, elicit TRE-like responses thus conferring TPA responsivity to the CRE site.[86] Further upstream into the 5′ flanking region of the hCRH gene, eight perfect consensus AP-1 binding sites have been detected.[65] Their ability to mediate TPA directed enhancement of hCRH gene expression has not been tested by conventional reporter gene assays as yet. EGF, however, has been shown to stimulate ACTH secretion in the primate and to directly stimulate CRH secretion by rat hypothalamic organ cultures *in vitro*.[87]

*GRE (glucocorticoid response element)*: Glucocorticoids are the final effectors of the hypothalamic–pituitary–adrenal (HPA) axis and participate in the control of whole-body homeostasis and the organism's adaptive response to stress.[10,11] These hormones exert their effects through their cytoplasmic receptors. When in the ligand-unbound/inactive state, these receptors are in the form of a hetero-oligomer with a hsp90 dimer and other proteins.[88] The ligand-bound receptors dissociate from the hetero-oligomer, homodimerize and translocate into the nucleus, where they interact with DNA to transactivate appropriate hormone-responsive genes that contain the consensus sequence 5′-GGTA-

CANNNTGTTCT-3′, GRE, in their 5′ flanking regions.[89,90] The activated receptors also interact at the protein level with the c-jun component of the AP-1 transcription factor, preventing this factor from exerting its effects on TRE/AP1-responsive genes.[91,92] Consistent with the structural features of its promoter, the glucocorticoid receptor (GR) gene is constitutively expressed in all or most tissues of the organism.[93–96] Cellular hsp90 content appears to be an important determinant of a cell's sensitivity to glucocorticoids and the interaction of hsp90 with the unliganded form of GR appears to be a modulator of the glucocorticoid signal transduction cascade.[97–102]

Glucocorticoids play a key regulatory role in the biosynthesis and release of CRH.[11,103] Glucocorticoids down-regulate rat and ovine hypothalamic CRH peptide and mRNA levels.[61,104] Stably introduced hCRH gene in AtT-20 cells is also subject to negative glucocorticoid regulation.[105] Adrenalectomy and dexamethasone administration in the rat elicits differential CRH mRNA responses in the PVN and the cerebral cortex, stimulating and suppressing it respectively in the former, but not influencing it in the latter.[106] Glucocorticoids can also stimulate hCRH gene expression in other tissues such as the human placenta[107] and the central nucleus of the amygdala.[108] A construct containing the proximal 900 bp of the 5′ flanking region of the hCRH gene coupled to a CAT reporter has been transiently expressed in COS cells and found to confer negative and positive glucocorticoid effects, depending on the cotransfection of a GRcDNA expression plasmid.[86] The molecular mechanism by which glucocorticoids regulate hCRH gene expression is somewhat obscure. Glucocorticoid suppression of hCRH gene expression might be mediated by the inhibitory interaction of the activated GR with the c-jun component of the AP-1 complex. On the other hand, glucocorticoid enhancement of hCRH gene expression might be mediated by the potentially active half perfect GREs present in the 5′ flanking region of the gene.[86] Half GREs have been shown to confer delayed secondary glucocorticoid responses to another gene.[109,110] Direct experimental demonstration would be required to substantiate the functionality of the 1/2 GREs present in the 5′ flanking region of the hCRH gene.

*ERE (estrogen response element)*: Gonadal steroids are the final effectors of the hypothalamic–pituitary–gonadal (HPG) axis. They control reproduction and sexual dimorphic physiology and behavior.[111] Androgens and estrogens ($E_2$) act by specific receptor-mediated processes to modulate the expression of genes with GRE or ERE (5′-GGTCANNNTGACC-3′) consensus sequences in their 5′ flanking regions, respectively.[90] The two ERE binding domains are exact palindromes, and half palindromic EREs can also mediate $E_2$ enhancement of gene expression.[112] Unlike the GR, androgen and estrogen receptors (ERs) are not constitutively expressed and have a narrower tissue distribution.[111,113,114] ER, although not studied as well as GR, appears to have similar hsp90-dependent activational characteristics.[100,115] It interacts with the c-jun and c-fos components of the AP1 binding proteins and thus also regulates gene expression from TRE sites in a negative or positive fashion.[116,117] It is not known whether 1/2 GREs may in some cases confer weak androgen receptor-mediated transactivation by analogy with the weak and delayed GR transactivation by glucocorticoids via the 1/2 GRE enhancer.[109,110] Some regions of the male brain possess aromatase activity to convert androgens to estrogens, which may then modulate CNS functions through the more commonly expressed ERs in brain areas of both sexes.[111]

Human female hypothalami have higher concentrations of CRH than the male ones.[118] Chronic estradiol treatment of ovariectomized rats stimulates PVN CRH peptide levels[119] and increases ACTH and corticosterone secretion basally and in response to stress.[120] In addition, rat PVN CRH mRNA levels increase in the afternoon of proestrous, at the approximate time of the $E_2$-induced preovulatory surge of LH.[121] These findings indicate that gonadal steroids may have an effect on the CRH-secreting neuron and suggest bidirectional interactions between the HPA and HPG axes through their final effectors. A direct $E_2$ enhancement of the CAT reporter was found by using two overlapping hCRH 5′ flanking region-driven CAT constructs in transient expression assays. Furthermore, the two perfect half palindromic EREs present in the common area of both CRH constructs, bound specifically to a synthetic peptide spanning the DNA binding domain of the human ER.[122] These findings demonstrate that hCRH gene expression is under direct $E_2$ regulation.

*TATA*: The TATA box is a highly conserved structural feature, present –20 to –30 from the transcription start site of many genes. This element appears to position the transcription start site by eukaryotic polymerase II, and participates in transcriptional control of gene expression.[123] Most genes that do not have a TATA box in their promoter region are constitutively expressed and have multiple transcription initiation sites.[95] The hCRH gene has two proximal TATA boxes located at –30 and –195 in its 5′ flanking region

(Fig. 2). The promoter-like properties of the more distal TATA box were studied *in vitro* after deletion of the more proximal TATA box.[86] These studies demonstrated that the −195 TATA box was active in initiating transcription and was responsive to cAMP, TPA and glucocorticoids.

The majority of hCRH transcripts in most tissues and cell lines studied initiate at +1. However, transcripts starting at −163 and −130 have also been detected in some tissues and cell lines, suggesting that the −195 TATA box is transcriptionally active *in vivo* in a variety of sites of CRH expression, contributing up to 30–40% of the total pool of cytoplasmic hCRH mRNA.[80,124] Additional upstream, potentially active start sites are present in this gene, and the ratio of transcripts initiating at such sites might also be tissue-specific.[60] Regarding the physiological significance of the longer transcripts, it was proposed that these might have a higher degree of secondary structure and might be more stable and long-lived than the short ones.[86]

TATA boxes have also been implicated in transcriptional regulation by the p53 growth suppressor gene. More specifically, p53 appears to control cellular activity by suppressing the transcription of genes with a TATA box in their promoter region through direct interaction.[125] The region −1.7 to −3.3 kb flanking the hCRH gene has a total of eight additional TATA boxes, which might also interact with p53 and influence CRH gene expression.[65]

*Other elements:* Tissue-specific and other elements, potentially unique to the CRH gene, may be involved in the control of its expression. Their identification will require detailed analysis by both conventional and transgenic functional assays, and their characterization will provide a better understanding of the control of the HPA axis.

### Tissue-specific and stress-related expression of the hCRH gene

As discussed above, the CRH gene is widely expressed throughout the body, suggesting that its product may have autocrine or paracrine actions. Strong evidence for the presence of tissue-specific enhancers in the human and primate CRH gene is its expression in the placenta and decidua.[126] Such expression is absent in the placenta of rodents,[127] suggesting that these enhancers may be carried in a segment of the regulatory region of the gene potentially subject to rearrangement in the non-expressing species.[65] Alternatively, the presence of tissue-specific repressor sequences in rodents may account for the observed differences in placental expression of the CRH gene between these species and primates.

Differential distribution of short and long hCRH mRNA transcripts has been detected in several tissues and under varying physiological conditions.[80,124] Tissue-specific and/or stress-dependent differential utilization of the two hCRH promoters, may explain these observations. Differential mRNA stability would then be a particularly important feature in CRH homeostasis, primarily in conditions of chronic stress, since in the latter case sustained production of CRH would be required and the long stable mRNAs produced by activation of the distal promoter would be beneficial to the organism.[86]

### Harmony in antithesis. Hormonal regulation of the hCRH gene

Apparently, depending on its site of expression, the hCRH gene responds antithetically to glucocorticoids.[65,77,107,108] The antithetical glucocorticoid effects on a hCRH promoter-drive CAT construct may be explained by the stimulation of the half GREs by high levels of ligand-bound GR, which presumably override the blockade of transcription exerted by lower levels of the activated GR through interaction with cjun-cfos, as discussed above. Is this mechanism physiologically relevant, particularly in view of the constitutive expression of the GR gene? Potentially yes, especially if one takes into account the heat shock proteins (hsp).

Although the GR content of various tissues is similar,[101,113] their sensitivity to glucocorticoids may vary substantially,[125] suggesting that some other cellular factor(s) is the principal modulator of glucocorticoid effect. There is a high tissue-specific fluctuation of hsp90 supporting a correlation between tissue hsp90 content and the sensitivity of this tissue to glucocorticoids.[101] For instance, tissues with high hsp90 content appear to be quite sensitive to glucocorticoids in contrast with tissues with a low hsp90 content,[101,128] and as hsp90 content increases one would expect a parallel increase of the effective concentration of tissue GR.[97,101] The relatively small fluctuation of hsp90 levels during immobilization stress,[102] on the other hand, suggests that the other proteins of the unbound GR hetero-oligomer[99] might also participate in the control of the sensitivity of tissues to glucocorticoids during stress.[101] Very little is known about the tissue-specific expression and the regulation of these proteins during stress, inflammation or debilitating disease. A proposed model summarizing these observations is shown in Fig. 3. This model

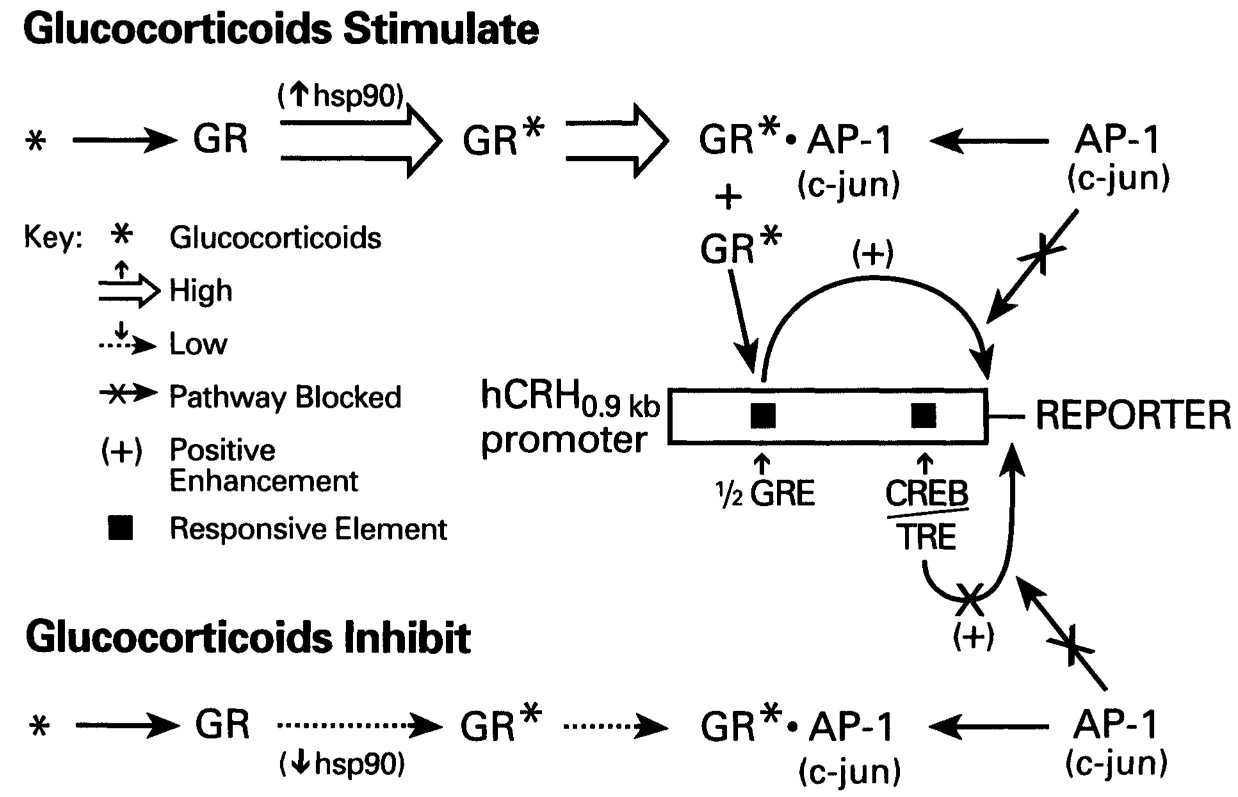

FIG. 3. A proposed model explaining the mechanism of tissue-specific antithetical effects of glucocorticoids on hCRH gene expression. In this model it is assumed that basal expression of the hCRH gene is under constant AP-1 enhancement. Elevation of ambient glucocorticoid levels may elicit activation of GR to different degrees, depending on the tissue hsp90 content.[101] Stimulation of hCRH gene expression would be predicted in tissues with high hsp90 content, such as testis, thymus or pineal, mediated by excess activation of GR that bypasses the AP-1 block and enhances transcription through the 1/2 GRE element. Inhibition would be expected in tissues with a low hsp90 content such as liver, spleen or pituitary, mediated by suppression of AP-1 enhancement through inactive complex formation with activated GR. Immunophilins, hsp70 and other factors interacting with the GR/hsp90 complex[99] may also modulate the activation of GR.

introduces a hypothetical general mechanism to account for the differential sensitivity and direction of effects of various tissues to glucocorticoids. This mechanism involves genes that are regulated by both the growth-promoting AP1 factors and by the differentiation-promoting, antigrowth glucocorticoid hormones. Since the hCRH gene contains both types of enhancers in its promoter region, it may potentially respond as outlined in Fig. 3. The mechanism proposed in Fig. 3 may also explain the frequently observed antithetical effects of chronic glucocorticoid administration in clinical practice.

### Potential implications of CRH gene regulation for the sexual dimorphism of the stress response and the immune/inflammatory reaction

Both the stress response and the immune/inflammatory reaction are associated with sexual dimorphism, both being more robust in the female or castrated male than in the intact male. The basis of this dimorphism may be gonadal steroid regulation of the components of the stress response. The demonstration of direct $E_2$-effects on hCRH gene expression,[122] implicates the CRH gene and, therefore, the HPA axis, as a potentially important target of ovarian steroids and a potential mediator of gender-related differences in the stress response and HPA axis activity. These effects of $E_2$ on the CRH neuron suggest that the HPG axis, which is known to be inhibited by hormones of the HPA axis at the hypothalamic, pituitary, gonadal and sex steroid target tissue levels during stress,[12,129] also appears to influence the latter in a positive fashion, by slightly enhancing CRH gene transcription. Thus, these data support a mutual, bidirectional interaction between the HPG and HPA axes, as depicted heuristically in Fig. 4.

The slightly enhanced CRH neuron activation by estrogen may not only explain why normal women have a slightly higher ACTH response to oCRH than normal men, as well as a slightly decreased ability of the glucocorticoid negative

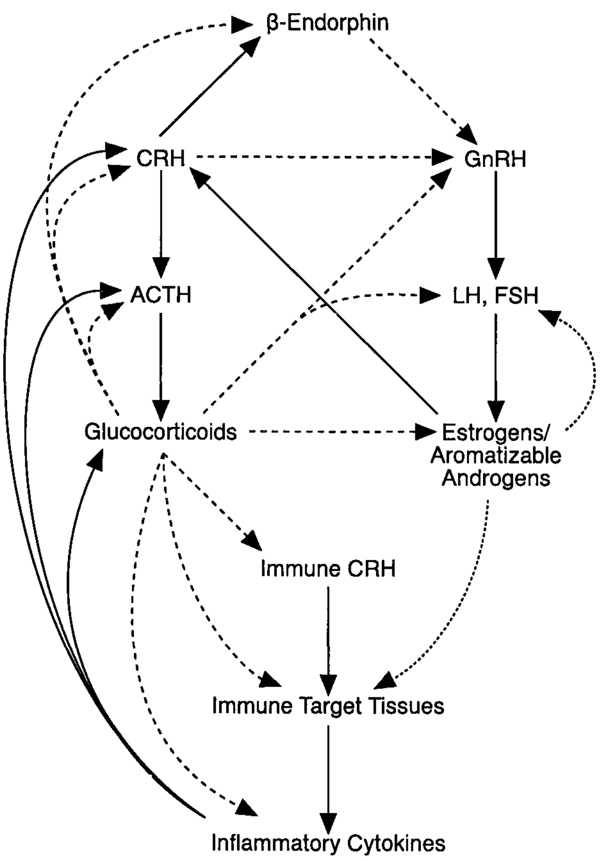

FIG. 4. A hypothetical model of the interactions between the HPG and HPA axes and the immune/inflammatory response. Solid lines indicate stimulation, broken lines inhibition, and the dotted lines conditional inhibition or stimulation. This model suggests that the interactions between these axes are not unidirectional, with the HPA axis inhibiting the HPG axis at multiple levels, as reviewed,[12] but bidirectional, with estrogen potentially stimulating both the CRH neuron, and therefore, the HPA axis, the peripheral production of immune CRH, and, hence, the immune/inflammatory response.[122] Immune CRH has been shown to exert proinflammatory effects *in vitro* and *in vivo*, including enhanced production of cytokines and other mediators of inflammation, which in turn stimulate hypothalamic production of CRH, pituitary production of ACTH and adrenal production of glucocorticoids. Although not included in the figure, gonadal steroid/cytokine interactions have been demonstrated for IL-6 actions.[160]

feedback to shut off the ACTH and cortisol responses,[129] but may also provide an explanation as to why various emotional disorders characterized by elevated CRH secretion,[12] such as depression and anxiety, have a higher incidence in women than in men.[130] Also, the same findings may explain why puberty/adolescence, the postpartum and the perimenopausal period, during all of which marked changes in estrogen production take place, are characterized by increased incidence of emotional disorders.

In addition to explaining the slightly increased, basal and stress stimulated HPA axis function in the female gender, the $E_2$-induced enhancement of the CRH neuron may also help explain the paradox of the negative $E_2$ feedback effect on the GnRH neuron,[131] which, unlike the PVN, lacks ERs.[132] The negative $E_2$ feedback might, thus, be exerted indirectly, via a subgroup of CRH neurons. CRH has been reported to suppress GnRH secretion through both a direct and an indirect, arcuate nucleus POMC/β-endorphin-mediated path.[12,133]

The immune/inflammatory reaction is greater in female than in male animals and humans, and in keeping with this, autoimmune inflammatory disease has a significantly higher prevalence in the female than the male sex of several species.[134–136] Estrogens, generally, have been shown to activate some components of the immune/inflammatory reaction, while androgens suppress it.[136–138] Markedly elevated secretion of immune CRH in various inflammatory sites has been demonstrated in the Lewis rat,[44,47] an animal model of increased susceptibility to autoimmune inflammatory disease, in which decreased hypothalamic CRH secretion and, hence, diminished glucocorticoid production and defective suppression of inflammation has been demonstrated.[139,140] Although the decreased production of CNS CRH and increased secretion of immune CRH are associated with the high susceptibility of this animal to autoimmune inflammatory disease in both sexes, both the susceptibility and the actual inflammatory responses, including expression of immune CRH in peripheral inflammatory sites, are greater in the female than the male.[137,138] $E_2$-mediated enhancement of immune CRH secretion might be a partial explanation for this sexual dimorphism in the Lewis rat, as well as, albeit to a lesser extent, in other rat strains or animal species.[141]

Inflammatory sites, such as the athritic joints of patients with rheumatoid arthritis, contain high levels of immunoreactive CRH in the synovial fluid and inside cultured synoviocytes.[48] Interestingly, patients with rheumatoid arthritis have been shown to have poor or deficient responses of their HPA axes to the stress of major surgery, when compared with patients with osteoarthritis having similar surgery.[142] Rheumatoid arthritis patients also have inappropriately normal or low normal basal diurnal concentrations of plasma cortisol[142,143] making them strictly analogous to the Lewis rat model of autoimmune/inflammatory disease.[139,140]

The above studies suggest that homeostatic regulation involves complex mutual interactions between the reproductive axis, HPA axis and the immune system, in which $E_2$ and CRH may play central roles (Fig. 4). Certainly, other molecules involved in the regulation of these axes, such as several neurotransmitters, cytokines and lipid mediators, also participate in the above interactions and may contribute to their sexual dimorphism.[144] Thus, a neurotransmitter, serotonin, has been shown to stimulate both CRH and ACTH secretion.[145,146] The inflammatory cytokines tumour necrosis factor-α,[147,148] interleukin-1[148–150] and interleukin-6,[148,149,151] have been shown to activate acutely hypothalamic CRH secretion and, more chronically, pituitary ACTH[147,152,153] and, perhaps, glucocorticoid secretion,[151,152] whereas several lipid mediators of inflammation cause CRH, ACTH and glucocorticoid secretion.[150,154–157] These findings explain the original pioneering studies, in which CRH-, ACTH- and glucocorticoid-releasing bioactivities were found in the serum or supernatants of stimulated immunocytes.[155,158,159] Thus, immune CRH, by participating in the regulation of the immune response at the level of the leukocyte, may be also viewed as a peripheral coordinator of immune–neuroendocrine interactions.

### References

1. Harris GW. Neural control of the pituitary gland. *Physiol Rev* 1948; **28**: 139.
2. Guillemin R, Rosenberg B. Humoral hypothalamic control of anterior pituitary: a study with combined tissue cultures. *Endocrinol* 1955; **57**: 599–607.
3. Saffran M, Schally AV. The release of corticotropin by anterior pituitary tissue *in vitro*. *Can J Biochem Physiol* 1955; *33*: 408–415.
4. Vale W, Spiess J, Rivier C, Rivier J. Characterization of a 41-residue ovine hypothalamic peptide that stimulates secretion of corticotropin and β-endorphin. *Science* 1981; **213**: 1394–1397.
5. Vale W, River C, Brown MR, *et al.* Chemical and biological characterization of corticotropin releasing factor. *Recent Prog Horm Res* 1983; **39**: 245–270.
6. Antoni FA. Hypothalamic control of adrenocorticotropin secretion: advances since the discovery of 41-residue corticotropin factor. *Endocr Rev* 1986; **7**: 351–378.
7. Feek CM, Marante DJ, Edwards CRW. The hypothalamic–pituitary–adrenal axis. *Clin Endocr Metab* 1983; **12**: 597–618.
8. Axelrod J, Reisine TD. Stress hormones: their interaction and regulation. *Science* 1984; **224**: 452–459.
9 Rivier C, Vale W. Modulation of stress-induced ACTH release by corticotrophin-releasing factor, catecholamines and vasopressin. *Nature* 1983; **305**: 325–327.
10. Owens MJ, Nemeroff CB. Physiology and pharmacology of corticotropin-releasing factor. *Pharmacological Rev* 1991; **43**: 425–473.
11. Orth DN. Corticotropin-releasing hormone in humans. *Endocr Rev* 1992; **13**: 164–191.
12. Chrousos GP, Gold PW. The concepts of stress and stress system disorders: overview of physical and behavioral homeostasis. *JAMA* 1992; **267**: 1244–1252.
13. Stenzel-Poore MP, Heldwein KA, Stenzel P, Lee L, Vale WW. Characterization of the genomic corticotropin-releasing factor (CRF) gene from *Xenopus laevis*. Two members of the CRF family exist in amphibians. *Mol Endocrinol* 1992; **6**: 1716–1724.
14. Montecucchi PC, Heushen A. Amino acid composition and sequence analysis of sauvagine, a new active peptide from the skin of *Phyllomedusa sauvagei*. *Int J Peptide Protein Res* 198; **18**: 113–120.
15. Lederis K, Letter A, McMaster D, Moore G. Complete amino acids sequence of urotensin-1, a hypotensive and corticotropin-releasing neuropeptide from *Catostomus*. *Science* 1982; **218**: 162–164.
16. Kataoka H, Troetshler RG, Li TP, Kramer SJ, Comey RL, Schooley DA. Isolation and identification of a diuretic hormone from the tobacco hornworm, *Manduca sexta*. *Proc Natl Acad Sci USA* 1989; **86**: 2976–2980.
17. Blackburn MB, Kingan TG, Bodnarw, *et al.* Isolation and identification of a new diuretic peptide from the tobacco hornworm, *Manduca sexta*. *Biochem Biophys Res Commun* 1991; **81**: 927–932.
18. Rivier C, Rivier J, Lederis K, Vale W. *In vitro* and *in vivo* ACTH-releasing activity of ovine CRF, sauvagine and urotensin-1. *Regul Peptides* 1983; **5**: 139–143.
19. Lenz HJ, Fisher LA, Vale WW, Brown MR. Corticotropin-releasing factor, sauvagine and urotensin 1: effects on blood flow. *Am J Physiol* 1985; **249**: R85–R90.
20. Potter E, Behan DP, Fischer WH, Linton EA, Lowry PJ, Vale WW. Cloning and characterization of the cDNAs for human and rat corticotropin-releasing factor-binding proteins. *Nature* 1991; **349**: 423–426.
21. Potter E, Behan DP, Linton EA, Lowry PJ, Sawchenko PE, Vale WW. The central distribution of a corticotropin-releasing factor (CRF)-binding protein predicts multiple sites and modes of interaction with CRF. *Proc Natl Acad Sci USA* 1992; **89**: 4192–4196.
22. Vamvakopoulos NC, Sioutopoulou TO, Durkin S, Nierman W, Washmuth J, McPherson J. Mapping the human corticotropin releasing hormone binding protein gene (CRHBP) to the long arm of chromosome 5 (5q11.2–q13.3). *Genomics* 1995; **25**: 325–327.
23. Sobel DO. Role of cyclic AMP in corticotropin-releasing factor mediated ACTH release. *Peptides* 1985; **6**: 591–595.
24. Taylor AL, Fishman LM. Corticotropin-releasing hormone. *N Eng J Med* 1988; **319**: 213–222.
25. Abou-Samra AB, Harwood JP, Catt KJ. Mechanisms of action of CRF and other regulators of ACTH release in pituitary corticotrophs. *Ann NY Acad Sci* 1987; **512**: 67–84.
26. Grino M, Guillaume V, Boudouresque F, Margioris AN, Grisoli F, Jaquet P, Oliver C, Conte-Devolx B. Characterization of corticotropin-releasing hormone receptors on human pituitary corticotroph adenomas and their correlation with endogenous glucocorticoids. *J Clin Endocrinol Metab* 1988; **67**: 279–283.
27. Smith AI, Funder JE. Proopimelanocortin processing in the pituitary, central nervous system, and peripheral tissues. *Endocr Rev* 1988; **9**: 159–179.
28. Boutillier AL, Sassone-Gorsi P, Loeffler JP. The protoncogene c-fos is induced by corticotropin-releasing factor and stimulates pro-opiomelanocortin gene transcription in pituitary cells. *Mol Endocrinol* 1991; **5**: 1301–1310.
29. Herman JP, Schafer MK-H, Thompson RC, Watson SJ. Rapid regulation of corticotropin releasing hormone gene *in vivo*. *Mol Endocrinol* 1992; **6**: 1061–1069.
30. Chen R, Lewis KA, Perrin MH, Vale WW. Expression cloning of a human corticotropin-releasing-factor receptor. *Proc Natl Acad Sci USA* 1993; **90**: 8967–8971.
31. Vita N, Laurent P, Lefort S, *et al.* Primary structure and functional expression of mouse pituitary and human brain corticotrophin releasing factor receptors. *FEBS Let* 1993; **335**: 1–5.
32. Vamvakopoulos, NC, Sioutopoulou, TO. Human corticotropin releasing hormone receptor gene (CRHR) is located on the long arm of chromosome 17 (17q12–qter). *Chromosome Res* 1994; **2**: 471–473.
33. Perrin MH, Donaldson CJ, Chen R, Lewis KA, Vale WW. Cloning and functional expression of a rat brain corticotropin releasing factor (CRF) receptor. *Endocrinol* 1993; **133**: 3058–3061.
34. Rivier J, Rivier C, Vale W. Synthetic competitive antagonist of corticotropin-releasing factor: effect on ACTH secretion in the rat. *Science* 1984; **224**: 889–891.
35. DeSouza EB, Insel TH, Perrin MH, Rivier J, Vale WW, Kuhar MJ. Corticotropin releasing factor receptors are widely distributed within the rat central nervous system: an autoradiographic study. *J Neurosci* 1985; **5**: 3189–3203.
36. Parkes D, Rivest S, Lee S, Rivier C, Vale W. Corticotropin-releasing factor activates c-fos, NGFI-B, and corticotropin-releasing factor gene expression within the paraventricular nucleus of the rat hypothalamus. *Mol Endocrinol* 1993; **7**: 1357–1367.
37. Sutton RE, Koob GF, LeMoal M, Rivier J, Vale W. Corticotropin releasing factor produces behavioral activation in rats. *Nature* 1982; **297**: 331–333.
38. Brown MR, Fisher LA, Speiss J, Rivier C, Rivier J, Vale W. Corticotropin-releasing factor: actions on the sympathetic nervous system and metabolism. *Endocrinol* 1982; **111**: 928–931.
39. Sirinathsinghji DJH, Rees LH, Rivier J, Vale W. Corticotropin-releasing factor is a potent inhibitor of sexual receptivity in the female rat. *Nature* 1983; **305**: 232–235.
40. Lenz HG. Extrapituitary effects of corticotropin-releasing factor. *Horm Metab Res Suppl* 1987; **16**: 17–23.
41. Rivier C, Rivier J, Vale W. Stress-induced inhibition of reproductive functions: role of endogenous corticotropin-releasing factor. *Science* 1989; **231**: 607–609.
42. Dunn AJ, Berridge CW. Physiological and behavioural responses to corticotropin-releasing factor administration: is CRF a mediator of anxiety or stress response? *Brain Res Rev* 1990; **15**: 71–100.
43. Petraglia, F, Sawchenko PE, Rivier J, Vale W. Evidence for local stimulation of ACTH secretion by corticotropin-releasing factor in human placenta. *Nature* 1987; **328**: 717–719.
44. Ulisse S, Fabbri A, Dufau ML. Corticotropin-releasing factor receptors and actions in rat Leydig cells. *J Biol Chem* 1989; **264**: 2156–2163.

45. Fabbri A, Tinajero JC, Dafau ML. Corticotropin-releasing factor is produced by rat Leydig cells and has a major local antireproductive role in the testis. *Endocrinol* 1990; **127**: 1541–1543.
46. Karalis C, Sano J, Redwine J, Listwak S, Wilder R, Chrousos GP. Autocrine or paracrine inflammatory actions of corticotropin releasing hormone *in vivo*. *Science* 1991; **254**: 421–423.
47. Crofford LJ, Sano H, Karalis C, *et al.* Local secretion of corticotropin releasing hormone in the joints of Lewis rats with inflammatory arthritis and patients with rheumatoid arthritis. *J Clin Invest* 1992; **90**: 2555–2564.
48. Crofford LJ, Sano H, Karalis K, *et al.* Corticotropin-releasing hormone in synovial fluids and tissues of patients with rheumatoid arthritis and osteoarthritis. *J Immunol* 1993; **141**: 1–10.
49. Kavelaars A, Ballieux RE, Heijnen CJ. The role of IL-1 in the corticotropin-releasing factor and arginine vasopressin-induced secretion of immunoreactive β-endorphin by human peripheral blood mononuclear cells. *J Immunol* 1989; **142**: 2338–2342.
50. Christine Leu S-J, Singh VK. Stimulation of interleukin-6 production by corticotropin-releasing factor. *Cell Immunol* 1992; **143**: 220–227.
51. Angioni S, Petraglia F, Gallinelli A, *et al.* Corticotropin-releasing hormone modulates cytokine release in cultured human peripheral blood mononuclear cells. *Life Sci* 1993; **53**: 1735–1742.
52. Genedani S, Bernardi M, Baldini MG, Bertolini N. Influence of CRF and alpha-MSH on the migration of human monocytes *in vitro*. *Neuropeptides* 1992; **23**: 99–102.
53. Koshida H, Kotake Y. Corticotropin-releasing factor enhances the superoxide anion production of rabbit peritoneal macrophages stimulated with *N*-formyl-methionyl-leucyl-phenylalanine. *Life Sci* 1994; **54**: 539–543.
54. Muler OA, Stalla GK, von Werder K. Corticotropin releasing factor: a new tool for the differential diagnosis of Cushing's syndrome. *J Clin Endocrinol Metab* 1983; **57**: 227–229.
55. Chrousos GP, Shulte HM, Oldfield EH, Gold, PW, Cutler GB Jr, Loriaux DL. The corticotropin-releasing factor stimulation test. *N Engl J Med* 1984; **310**: 622–626.
56. Chrousos GP. Clinical applications of corticotropin-releasing factor. *Ann Intern Med* 1985; **102**: 344–358.
57. Habener JF. Genetic control of hormone formation, In: *Williams Textbook of Endocrinology 8th Ed.* Wilson J, Foster D (eds) Saunders Co. 1992; 9–33.
58. Reichlin S. Neuroendocrinology, In: *Williams Textbook of Endocrinology 8th Ed.* Wilson J, Foster D (eds) Saunders Co. 1992; 135–219.
59. Shibahara S, Morimoto Y, Furutani Y, *et al.* Isolation and sequence analysis of the human corticotropin-releasing factor precursor gene. *EMBO J* 1983; **2**: 775–779.
60. Thompson RC, Seasholtz AF, Herbert E. Rat corticotropin-releasing hormone gene: sequence and tissue-specific expression. *Mol Endocrinol* 1987; **1**: 363–370.
61. Roche RJ, Crawford RJ, Fernley RT, Tregear GW, Coghlan JP. Nucleotide sequence of the gene coding for ovine corticotropin-releasing factor and regulation of its mRNA levels by glucocorticoids. *Gene* 1988; **71**: 421–431.
62. Arbiser JL, Morton CC, Bruns GAP, Majzoub JA. Human corticotropin releasing hormone gene is located on the long arm of chromosome 8. *Cytogenet Cell Genet* 1988; **47**: 113–116.
63. Adler GK, Rosen LB, Fiandaca MJ, Majzoub JA. Protein kinase-C activation increases the quantity and poly(A) tail length of corticotropin-releasing hormone messenger RNA in NPLC cells. *Mol Endocrinol* 1992; **6**: 476–484.
64. Vamvakopoulos NC, Karl M, Mayol V, *et al.* Structural analysis of the regulatory region of the human corticotropin releasing hormone gene. *FEBS Lett* 1990; **267**: 1–5.
65. Vamvakopoulos NC, Chrousos GP. Structural organization of the 5′ flanking region of the human corticotropin releasing hormone gene. *DNA Sequence-J DNA Sequencing and Mapping* 1993; **4**: 197–206.
66. Deutsch PJ, Hoeffler JP, Jameson JL, Lin JC, Habener JF. Structural determinants for transcriptional activation by cAMP-responsive DNA elements. *J Biol Chem* 1988; **263**: 18466–18472.
67. Hoeffler JP, Deutsch PJ, Lin J, Habener JF. Distinct cyclic AMP and phorbol ester-mediated signal transduction pathways converge at the level of transcriptional activation of DNA-binding proteins. *Mol Endocrinol* 1989; **3**: 868–880.
68. Yamamoto KK, Gonzalez GA, Menzel P, Rivier J, Montminy MR. Characterization of bipartite activator domain in transcription factor CREB. *Cell* 1990; **60**: 611–617.
69. Lee CQ, Yun Y, Hoeffler JP, Habener JF. Cyclic-AMP-responsive transcriptional activation of CREB-327 involves interdependent phosphorylated subdomains. *EMBO J* 1990; **9**: 4455–4465.
70. Sheng M, Thompson MA, Greenberg ME. CREB: a Ca-regulated transcription factor phosphorylated by calmodulin dependent kinases. *Science* 1991; **252**: 1227–1230.
71. Hoeffler JP, Meyer TE, Waeber G, Habener JF. Multiple cyclic adenosine 3′,5′-monophosphate response element DNA-binding proteins generated by gene diversification and alternative exon splicing. *Mol Endocrinol* 1990; **14**: 920–930.
72. Waeber G, Meyer TE, Lesieur M, Hermann HL, Gerard N, Habener JF. Developmental stage-specific expression of the cyclic AMP response element binding protein CREB during spermatogenesis involves alternative exon splicing. *Mol Endocrinol* 1991; **5**: 1418–1430.
73. Landschultz WH, Johnson PF, Adashi EY, Graves BJ, McKnight SL. Isolation of a recombinant copy of the gene encoding C/EBP. *Genes Dev* 1988; 32: 786–800.
74. Hattori K, Angel P, LeBeau MM, Karin M. Structure and chromosomal localization of the functional intronless human Jun protooncogene. *Proc Natl Acad Sci USA* 1988; **85**: 9148–9152.
75. Hai T, Curran T. Cross-family dimerization of transcription factors Fos/Jun and ATF/CREB alters DNA binding specificity. *Proc Natl Acad Sci USA* 1991: **88**: 3720–3724.
76. deGroot RP, Sassone-Gorsi P. Hormonal control of gene expression: multiplicity and versatility of cyclic adenosine 3′,5′-monophosphate-responsive nuclear regulators. *Mol Endocrinol* 1993; **7**: 145–153.
77. Emanuel RL, Girard DM, Thull DL, Majzoub JA. Second messengers involved in the regulation of corticotropin-releasing hormone mRNA and peptide in cultured rat fetal hypothalamic primary cultures. *Endocrinol* 1990; **126**: 3016–3021.
78. Dorin RI, Takahashi H, Nakai Y, Fukata J, Naitoh Y, Imura H. Regulation of human corticotropin-releasing hormone gene expression by 3′,5′-cyclic adenosine monophosphate in a transformed mouse corticotroph cell line. *Mol Endocrinol* 1989; **3**: 1537–1544.
79. Van LP, Spengler DH, Holsboer F. Glucocorticoid repression of 3′-5′-cyclic-adenosine monophosphate-dependent human corticotropin-releasing-hormone gene promoter activity in a transfected mouse anterior pituitary cell line. *Endocrinol* 1990; **127**: 1412–1418.
80. Adler GK, Smas CM, Fiandaca M, Frim DM, Majzoub JA. Regulated expression of the human corticotropin releasing hormone gene by cyclic AMP. *Mol Cell Endocrinol* 1990; **70**: 165–174.
81. Seaholtz AF, Thompson RC, Douglass JO. Identification of a cyclic adenosine monophosphate-responsive element in the rat corticotropin-releasing hormone gene. *Mol Endocrinol* 1988; **2**: 1311–1319.
82. Spengler D, Rupprecht R, Van LP, Holsboer F. Identification and characterization of a 3′,5′-cyclic adenosine corticotropin-releasing hormone gene promoter. *Mol Endocrinol* 1992; **6**: 1931–1941.
83. Angel P, Imagawa M, Chiu R, *et al.* Phorbol ester-inducible genes contain a common cis element recognized by a TPA-modulated trans acting factor. *Cell* 1987; **49**: 729–739.
84. Radler-Pohl A, Gebel S, Sachsenmaier C, *et al.* The activation and activity control of AP-1 (Fos/Jun). *Ann NY Acad Sci* 1993; **684**: 127–148.
85. Karin M. Too many transcription factors: positive and negative interactions. *New Biologist* 1990; **2**: 126–131.
86. Vamvakopoulos NC, Chrousos GP. Regulated activity of the distal promoter-like element of the human corticotropin releasing hormone gene and secondary structural features of its corresponding transcripts. *Mol Cell Endocrinol* 1993; **94**: 73–78.
87. Luger A, Calogero A, Kalogeras K, *et al.* Interaction of epidermal growth factor with the hypothalamic–pituitary–adrenal axis: potential phsyiologic relevance. *J Clin Endocrinol Metab* 1987; **66**: 334–337.
88. Dalman FC, Scherrer LC, Taylor LP, Akil H, Pratt WB. Localization of the 90-kDA heat shock protein binding site within the hormone binding domain of the glucocorticoid receptor by peptide recognition. *J Biol Chem* 1991; **266**: 3482–3490.
89. Yamamoto KR. Steroid receptor regulated transcription of specific genes and gene networks. *Annu Rev Genet* 1984; **19**: 209–252.
90. Beato M. Gene regulation by steroid hormones. *Cell* 1989; **56**: 335–344.
91. Diamond MI, Miner JN, Yoshinaga SK, Yamamoto KR. Transcription factor interactions: selectors of positive or negative regulation from a single DNA element. *Science* 1990; **249**: 1266–1272.
92. Truss M, Beato M. Steroid hormone receptors: Interaction with deoxyribonucleic acid and transcription factors. *Endo Rev* 1993; **14**: 459–479.
93. Kalinyak JE, Dorin RI, Hoffman AR, Perlman AJ. Tissue-specific regulation of glucocorticoid receptor mRNA by dexamethasone. *J Biol Chem* 1987; **262**: 10441–10444.
94. Kalinyak, JE, Griffin CA, Hamilton RW, Bradshaw JG, Perlman AJ, Hoffman AR. Developmental and hormonal regulation of glucocorticoid receptor messenger RNA in the rat. *J Clin Invest* 1989; **841**: 1843–1848.
95. Zong J, Ashraf J, Thompson EB. The promoter and first, untranslated exon of the human glucocorticoid receptor gene are GC rich but lack consensus glucocorticoid receptor element sites. *Mol Cell Biol* 1990; **10**: 5580–5585.
96. Vamvakopoulos NC, Mayol V, Margioris A, Chrousos GP. Lack of dexamethasone modulation of mRNAs involved in the glucocorticoid signal transduction pathway in two cell systems. *Steroids* 1992; **57**: 282–287.
97. Picard D, Khursheed B, Garabedian MJ, Fortin MG, Lindquist S, Yamamoto KR. Reduced levels of hsp90 compromise steroid receptor action *in vivo*. *Nature* 1990; **348**: 166–168.
98. Cadepond F, Chambraud BB, Jibard N, Schweizer-Groyer G, Segard-Maurel I, Baulieu EE. Interaction of glucocorticoid receptor and wild type or mutant 90-kDa heat shock protein coexpressed in baculovirus-infected Sf9 cells. *Proc Natl Acad Sci USA* 1993; **90**: 10434–10438.

99. Hutchison KA, Scherrer LC, Czar MJ, *et al.* Regulation of glucocorticoid receptor function through assembly of a receptor-heat shock protein complex. *Ann New York Acad Sci* 1993; **684**: 35–48.
100. Smith DF, Toft DO. Steroid receptors and their associated proteins. *Mol Endocrinol* 1993; **7**: 4–11.
101 Vamvakopoulos NC. Tissue-specific expression of heat shock proteins 70 and 90. Potential implication for differential sensitivity of tissues to glucocorticoids. *Mol Cell Endocrinol* 1993; **98**: 49–54.
102. Vanvakopoulos NC, Fukuhara K, Patchev V, Chrousos GP. Effect of single and repeated immobilization stress on the heat shock protein 70/90 system of the rat. Glucocorticoid-independent reversible reduction of hsp90 in the liver and spleen. *Neuroendocrinology* 1993; **57**: 1057–1065.
103. Munck A, Guyre PM, Holbrook NJ. Physiological functions of glucocorticoids in stress and their relation to pharmacological actions. *Endocr Rev* 1984; **5**: 25–44.
104. Jingami H, Matsukura S, Numa S, Imura H. Effects of adrenalectomy and dexamethasone administration on the level of prepro-corticotropin-releasing factor messenger ribonucleic acid (mRNA) in the hypothalamus and adrenocorticotropin/β-lipotropin precursor mRNA in the pituitary in rats. *Endocrinol* 1985; **117**: 1314–1320.
105. Adler GK, Smas CM, Majzoub JA. Expression and dexamethasone regulation of the human corticotropin-releasing hormone gene in a mouse anterior pituitary cell line. *J Biol Chem* 1988; **262**: 5846–5852.
106. Frim DM, Robinson BG, Pasieka KB, Majzoub JA. Differential regulation of corticotropin-releasing hormone mRNA in rat brain. *Am J Phys* 1990; **258** (Endocrinol Metab 21): E686–E692.
107. Robinson BG, Emanuel RL, Frim DM, Majzoub JA. Glucocorticoid stimulates expression of corticotropin-releasing hormone gene in human placenta. *Proc Natl Acad Sci USA* 1988; **85**: 5244–5248.
108. Swanson LW, Simmons DM. Differential steroid hormone and neuronal influence on peptide and mRNA levels in CRH cells of the paraventricular nucleus: a hybridization histochemical study in the rat. *J Comp Neurol* 1989; **285**: 413–435.
109. Chan GCK, Hess P, Meenakshi T, Carlsted-Duke J, Gustafsson J-A, Payvar F. Delayed secondary glucocorticoid response elements. *J Biol Chem* 1991; **266**: 22634–22644.
110. Slater EP, Hesse H, Muller JM, Beato M. Glucocorticoid receptor binding site in the mouse α-amylase 2 gene mediates response to the hormone. *Mol Endocrinol* 1993; **7**: 907–914.
111. Yen SSC, Jaffe RB. *Reproductive Endocrinology, 3rd ed.* Saunders Co. 1991. Philadelphia.
112. Kato S, Tora L, Yamauchi J, Masushige S, Bellard M, Chambon P. A far upstream estrogen response element of the ovalbumin gene contains several half-palindromic 5′-TGACC-3′ motifs acting synergistically. *Cell* 1992; **68**: 731–742.
113. Ballard PC, Baxter JD, Higgins SJ, Rousseau GG, Tomkins GM. General presence of glucocorticoid receptors in mammalian tissues. *Endocrinol* 1974; **94**: 998–1002.
114. Pelletier G, Liao N, Follea N, Govindan MV. Mapping of estrogen receptor producing cells in the rat brain by *in situ* hybridization. *Neurosci Lett* 1988; **94**: 23–28.
115. Pratt WB. At the cutting edge. Interaction of hsp90 with steroid receptors: organizing some diverse observations and presenting the newest concepts. *Mol Endocrinol* 1990; **74**: C69–C76.
116. Tzukerman M, Zhang X-K, Pfahl M. Inhibition of estrogen receptor activity by the tumor promoter 12-O-tetradecanoyphorbol-13-acetate: a molecular analysis. *Mol Endocrinol* 1991; **5**: 1983–1992.
117. Gaub MP, Bellard M, Schener I, Chambon P, Sassone-Corsi P. Activation of the ovalbumin gene by the estrogen receptor involves the Fos–Jun complex. *Cell* 1990; **63**: 1267–1276.
118. Frederiksen SO, Ekman R, Gottfries C-G, Widerlov E, Jonsson S. Reduced concentrations of galanin, arginine, vasopressin, neuropeptide Y and peptide YY in the temporal cortex but not in the hypothalamus of brains from schizophrenics. *Acta Psychiatr Scand* 1991; **83**: 273–277.
119. Haas DA, George SR. Gonadal regulation of corticotropin-releasing factor immunoreactivity in hypothalamus. *Brain Res Bull* 1988; **20**: 361–367.
120. Burgess LH, Handa RJ. Chronic-estrogen induced alterations inadrenocorticotropin and corticosterone secretion and glucocorticoid receptor-mediated functions in female rats. *Endocrinol* 1992; **131**: 1261–1269.
121. Bohler HCLJr, Zoeller RT, King JC, Rubin BS, Weber R, Merriam GR. Corticotropin releasing hormone mRNA is elevated on the afternoon of proestrous in the parvocellular paraventricular nuclei of the female rat. *Mol Brain Res* 1990; **8**: 259–262.
122. Vamvakopoulos NC, Chrousos, GP. Evidence of direct estrogenic regulation of human corticotropin releasing hormone gene expression: potential implications for the sexual dimorphism of the stress response and immune/inflammatory reaction. *J Clin Invest* 1993; **92**: 1896–1902.
123. Mitchell PJ, Tjian R. Transcriptional regulation in mammalian cells by sequence-specific DNA binding proteins. *Science* 1989; **245**: 371–378.
124. Dorin RI, Zlock DW, Kilpatrick K. Transcriptional regulation of human corticotropin releasing factor gene expression by cyclic adenosine 3′,5′-monophosphate: differential effects at proximal and distal promoter elements. *Mol Cell Endocrinol* 1993; **96**: 99–111.
125. Mack DH, Vartikar J, Pipas JM, Laiming LA. Specific repression of TATA-mediated but not initiator-mediated transcription by wold-type p53. *Nature* 1993; **363**: 281–283.
126. Grino M, Chrousos GP, Margioris AN. The corticotropin releasing hormone gene is expressed in human placenta. *Biochem Biophys Res Commun* 1987; **148**: 1208–1214.
127. Robinson BG, Arbiser HL, Emanuel RL, Majzoub JA. Species-specific placental corticotropin releasing hormone messenger RNA and peptide expression. *Mol Cell Endocrinol* 1989; **62**: 337–342.
128. Baxter JD, Forsham PH. Tissue effects of glucocorticoids. *Amer J Med* 1972; **53**: 573–589.
129. Rivier C, Vale W. Effect of the long term administration of corticotropin-releasing factor on the pituitary–adrenal and pituitary–gonadal axis in the male rat. *J Clin Invest* 1985; **75**: 689–694.
130. Galluci WT, Baum A, Laue L, *et al.* Sex differences in sensitivity of the hypothalamic–pituitary–adrenal axis. *Health Psychology* 1993; **12**: 420–425.
131. Yen SSC, Lein A. The apparent paradox of the negative and positive feedback control system on gonadotropin secretion. *Am J Obstet Gynecol* 1976; **126**: 942–954.
132. Shivers BD, Harlan RE, Morrell JI, Pfaff DW. Absence of estradiol concentration in cell nuclei of LHRH-immunoreactive neurons. *Nature* 1983; **304**: 345–347.
133. Ferin M, Van Vugt DA, Wardlaw SL. The hypothalamic control of the menstrual cycle and the role of endogenous opioid peptides. *Recent Prog Horm Res* 1984; **40**: 441–485.
134. Grossman CJ, Roselle GA, Mendehall, CL. Sex steroid regulation of autoimmunity. *J Steroid Biochem Mol Biol* 1991; **40**: 649–659.
135. Inman RD. Immunologic sex differences and the female predominance in systemic lupus erythematosus. *Arthritis Rheum* 1978; **21**: 849–852.
136. Raveche ES, Tjio JH, Boegel W, Steinberg AD. Studies of the effects of sex hormones on autosomal and X-linked genetic control of induced and spontaneous antibody production. *Arthritis Rheum* 1979; **22**: 1177–1187.
137. Wilder RL, Calandra GB, Garvin JA, Wright KD, Hansen CT. Strain and sex variation in the susceptibility to streptococcal cell wall-induced polyarthritis in the rat. *Arthritis and Rheumatism* 1982; **25**: 1064–1072.
138. Allen JB, Blatter D, Calandra GB, Wilder RL. Sex hormonal effects on the severity of streptococcal cell wall-induced polyarthritis in the rat. *Arthritis and Rheumatism* 1983; **26**: 560–563.
139. Sternberg EM, Hill HM, Chrousos GP, *et al.* Inflammatory mediator-induced hypothalamic–pituitary–adrenal axis activation is defective in streptococcal cell wall arthritis-susceptible Lewis rats. *Proc Natl Acad Sci USA* 1989; **86**: 2374–2378.
140. Sternberg EM, Scott Young W, Bernardini R, *et al.* A central nervous system defect in biosynthesis of corticotropin-releasing hormone is associated with susceptibility to streptococcal cell wall-induced arthritis in Lewis rats. *Proc Natl Acad Sci USA* 1989; **86**: 4771–4775.
141. Homo-Delarche F, Fitzpatrick F, Christoeff N, Nunez EA, Bach JF, Dardenne M. Sex steroids, glucocorticoids, stress and autoimmunity. *J Steroid Biochem Mol Biol* 1991; **40**: 619–637.
142. Chikanza IC, Petrou P, Chrousos GP, Kingsley G, Panayi GS. Defective hypothalamic response to immune/inflammatory stimuli in patients with rheumatoid arthritis. *Arthritis Rheum* 1992; **35**: 1281–1288.
143. Neeck G, Federiin K, Graef V, Rusch D, Schmidt K. Adrenal secretion of cortisol in patients with rheumatoid arthritis. *J Rheumatol* 1990; **17**: 24–29.
144. Imura H, Fukata JI, Mori T. Cytokines and endocrine function: an interaction between the immune and neuroendocrine systems. *Clin Endocrinol* 1991; **35**: 107–115.
145. Calogero AE, Bernardini R, Margioris AN, *et al.* Serotonin stimulates rat hypothalamic corticotropin releasing hormone secretion *in vitro. Peptides* 1989; **10**: 189–210.
146. Calogero AE, Bagdy G, Szemereti, Tartaglis ME, Gold PW, Chrousos GP. Mechanisms of serotonin agonist-induced activation of the hypothalamic–pituitary–adrenal axis in the rat. *Endocrinol* 1990; **126**: 1888–1894.
147. Bernardini R, Kamilaris TC, Calogero AE, Johnson EO, Gold PW, Chrousos GP. Interactions between tumor necrosis factor-α, hypothalamic corticotropin-releasing hormone and adrenocorticotropin secretion in the rat. *Endocrinol* 1990; **126**: 1876–1881.
148. Peristein RS, Whitnall MH, Abrams JS, Mougey EH, Neta R. Synergistic roles of interleukin-6, interleukin-1, and tumor necrosis factor in the adrenocorticotropin response to bacterial lipopolysacharide *in vivo. Endocrinol* 1993; **132**: 046–952.
149. Peristein RS, Mougey EH, Jackson WE, Neta R. Interleukin-1 and interleukin-6 act synergistically to stimulate the release of adrenocorticotropin hormone *in vivo. Lymphokine Cytokine Res* 1991; **10**: 141–146.
150. Bernardini R, Calogero A, Mauceri, Chrousos GP. Rat hypothalamic corticotropin releasing hormone secretion *in vitro* is stimulated by interleukin-1 in an eicosanoid-dependent manner. *Life Sci* 1990; **47**: 1601–1607.
151. Mastorakos G, Chrousos GP, Weber JS. Recombinant interleukin-6 activates the hypothalamic–pituitary–adrenal axis in humans. *J Clin Endocrinol Metab* 1993; **77**: 1690–1694.

152. Mazzocchi G, Musato FG, Malendowicz LK, Andreis PG, Nussdorfer GG. Interleukin-1β stimulates corticotropin-releasing hormone and adrenocorticotropin release by rat adrenal gland *in vivo*. *Mol Cell Neurosci* 1993; **4**: 267–270.
153. Spangelo BL, Judd AM, Isakson PC, MacLeod RM. Interleukin-6 stimulates anterior pituitary hormone release *in vitro*. *Endocrinol* 1989; **125**: 575–577.
154. Tominaga T, Fukata J, Naito Y, *et al.* Prostaglandin-dependent *in vivo* stimulation of adrenocortical steroidogenesis by interleukins. *Endocrinol* 1991; **128**: 562–531.
155. Witorsch RJ, Brodish A. Evidence for acute ACTH release by extrahypothalamic mechanisms. *Endocrinol* 1972; **90**: 1160–1170.
156. Bernardini R, Chiarenza A, Calogero AE, Gold PW, Chrousos GP. Arachidonic acid metabolites modulate rat hypothalamic corticotropin releasing hormone secretion *in vitro*. *Neuroendocrinol* 1989; **50**: 708–715.
57. Bernardini R, Calogero AE, Ehlich YH, Brucke T, Chrousos GP, Gold PW. The alkyl-ether phospholipid platelet-activating factor is a stimulator of the hypothalamic–pituitary–adrenal axis in the rat. *Endocrinol* 1989; **125**: 1067–1073.
158. Woloski BMRNJ, Smith EM, Meyer III EM, Fuller GM, Blalock JE. Corticotropin-releasing activity of monocytes. *Science* 1985; **230**: 1035–1037.
159. Whitcomb RW, Linehan WM, Wahl LM, Knazek, RA. Monocytes stimulate cortisol production by cultured human adrenocortical cells. *J Clin Endocrinol Metab* 1988; **66**: 33–38.
160. Poli V, Balena R, Fattori E, *et al.* Interleukin-6 deficient mice are protected from bone loss caused by estrogen depletion. *EMBO J* 1994; **13**: 1189–1196.

**Received 17 January 1995; accepted 3 March 1995**
